# Supplementary material for: Antimicrobial Residue Accumulation Contributes to Higher Levels of Rhodococcus equi Carrying Resistance Genes in the Environment of Horse-Breeding Farms
Source: Vet Sci. 2024 Feb 17;11(2):92. doi: 10.3390/vetsci11020092 (PMC10892917; doi:10.3390/vetsci11020092)
Supplement: Supplementary file 1 [file vetsci-11-00092-s001.zip › vetsci-2789981-supplementary.pdf]

Supplementary Materials:

Antimicrobial Standard	CAS-No.	Brand
2,5-Dichlorothiophene-3-sulfonamide	53595-68-9	Aldrich Chemistry
Ampicillin	69-53-4	Sigma-Aldrich
Azithromycin	117772-70-0	European Pharmacopeia
Clarithromycin	81103-11-9	European Pharmacopeia
Cycloheximide	66-81-9	VWR Life Science
Doxycycline hyclate	24390-14-5	U. S. Pharmacopeia
Erythromycin	114-07-8	U. S. Pharmacopeia
Tetracycline hydrochloride	64-75-5	Sigma-Aldrich
Tylosin	1401-69-0	Sigma-Aldrich

Table S1. List of antimicrobial standards used to create standard curves for mass spectrometry analysis. The antimicrobial concentrations used for the construction of standard curves were adjusted to the following gradient: 5 µg/mL, 1 µg/mL, 0.5 µg/mL, 0.1 µg/mL, 0.05 µg/mL, 0.01 µg/mL.
